# A Multi-Stage Framework for Refining Infant Daytime Sleep–Wake Labels from Wearable Accelerometer Data

**DOI:** 10.3390/s26134168

**Published:** 2026-07-02

**Authors:** Rama Krishna Thelagathoti, Vijaya Saraswathi Redrowthu, Danae Dinkel, Hesham H. Ali, Rohan M. Fernando

**Affiliations:** 1 Molecular Diagnostic Research Laboratory, Boys Town National Research Hospital, Omaha, NE 68131, USA; m.rohan.fernando@boystown.org; 2 Department of Computer Science and Engineering, VNR Vignana Jyothi Institute of Engineering and Technology, Hyderabad 500090, Telangana, India; vijayasaraswathi_r@vnrvjiet.in; 3 School of Health and Kinesiology, University of Nebraska Omaha, Omaha, NE 68182, USA; dmdinkel@unomaha.edu; 4 College of Information Science and Technology, University of Nebraska Omaha, Omaha, NE 68182, USA; hali@unomaha.edu

**Keywords:** actigraphy data analysis, infant sleep detection, label refinement, wearable accelerometers

## Abstract

**Highlights:**

- A novel Multi-Stage Sleep–Wake (MSW) framework was developed to refine infant sleep–wake annotations from noisy daytime accelerometer data.
- Models trained with MSW-derived labels achieve substantial performance gains (up to ~96% accuracy) relative to the refined labeling framework compared to those using parent-reported labels.
- Objective label refinement can effectively mitigate noise and bias in caregiver-reported sleep annotations.
- The proposed approach enables scalable and non-invasive infant sleep monitoring and sleep–wake annotation refinement wearable sensors.

**Abstract:**

Sleep is essential for infants’ physical and cognitive development. Unlike older children or adolescents, infants sleep longer durations, including multiple daytime naps. While nighttime sleep is easier to detect due to its extended periods, identifying daytime sleep is more challenging due to its short, fragmented nature and its similarity to idle wakefulness. Moreover, parent-reported sleep estimations are prone to error as continuous monitoring is often impractical. To address these limitations, we developed a Multi-Stage Sleep–Wake (MSW) classification approach using triaxial accelerometer data collected from wearable devices placed on infants’ ankles and waists over multiple days. This method systematically refines and classifies sleep and wake states through a series of analytical steps. This systematic process generates refined proxy sleep–wake labels that account for behavioral overlaps and correct mislabeled idle periods. We trained and validated multiple machine learning models using these labels and compared the results to parent annotated labels. Models trained using MSW-derived labels achieved 96.6% accuracy using Random Forest classifier compared to 72% using parent-reported labels. These findings demonstrate that the MSW framework produces a more consistent set of proxy sleep–wake annotations for model development, although the derived labels were not validated against an independent reference standard. Furthermore, the proposed MSW framework may serve as a practical label-refinement methodology for improving noisy caregiver-reported sleep annotations in future wearable-based infant sleep studies where independent sleep labels are unavailable.

## 1. Introduction

Sleep is essential for an infant’s cognitive, physical, and emotional development [1]. It supports brain maturation, memory consolidation, immune function, and overall growth [2,3]. Unlike older children and adolescents, infants have polyphasic sleep patterns, including multiple daytime naps in addition to nighttime sleep. According to the American Academy of Sleep Medicine (AASM), infants aged 4 to 12 months require 12 to 16 h of sleep per 24-h period, including both daytime and nighttime sleep [4,5,6]. On average, infants in their first-year sleep about 9–11 h at night and take 3–4 h of naps spread across the day [7]. These naps play a crucial role in maintaining mood regulation, attention span, and cognitive performance [8]. Failure to obtain adequate and appropriately timed naps has been linked to adverse developmental outcomes, such as impaired memory and behavioral problems [9].

Despite its importance, accurate sleep detection in infants remains a complex challenge. Traditional methods such as polysomnography (PSG) are considered the gold standard for sleep assessment, but they are invasive, require clinical settings, and often involve cumbersome equipment that is not suitable for infants [10,11]. Infants may not tolerate electrodes or restricted environments, which limits the feasibility of such techniques in naturalistic settings [12]. In contrast, behavioral observation by parents or caregivers—while less intrusive—requires continuous monitoring, which is impractical and subjective [13]. There is thus a critical need for non-invasive, objective methods that can reliably detect and quantify sleep in infants, particularly in real-world home environments.

Parent-reported activity diaries are frequently used as a proxy for sleep annotations, but they are inherently limited by recall bias and imprecise timing [14]. Caregivers may not be able to track sleep onset and wake times accurately, especially during fragmented or overlapping naps. For instance, if an infant sleeps from 9:45 am to 11:10 am, a parent might log the entire interval as “sleep until 11:30,” thereby introducing label noise and temporal inaccuracies. Additionally, diary reporting granularity (e.g., in 30-min blocks) fails to capture the nuanced transitions between sleep and wake states [15,16]. These challenges call for a more sophisticated and automated approach that can handle such inconsistencies and provide accurate sleep detection during the day, especially for naps that are shorter and harder to distinguish from idle wakefulness.

Accelerometer-based sensing has emerged as a promising, non-invasive tool for objective sleep–wake detection [17]. Triaxial accelerometers measure movement data in real time and have been validated for sleep monitoring in both adult and pediatric populations [18,19]. While numerous studies have explored their use in school-aged children and adolescents, fewer have focused on infants, who present unique challenges due to their frequent naps, subtle movements during sleep, and variability in behavioral states [20,21]. Devices worn on the ankle or waist allow continuous, unobtrusive monitoring of physical activity, providing a rich dataset to infer sleep–wake patterns [22,23]. However, the effectiveness of accelerometer-based sleep classification depends heavily on the quality of labels and the ability to distinguish between low-activity wake states and true sleep.

In this study, we collected triaxial accelerometer data from wearable devices placed on the ankle and waist of infants across multiple days in their natural home environment. Rather than relying solely on potentially noisy parent-reported sleep annotations, we developed MSW framework to systematically refine sleep–wake labels using objective movement patterns derived from accelerometer data. The proposed framework combines entropy-based thresholding, temporal smoothing, and idle wakefulness correction to generate more consistent proxy labels for daytime sleep annotation. The goals of the study were to: (1) develop an automated framework for refining daytime sleep and wake states from infant accelerometer data; (2) evaluate the impact of MSW-derived label refinement on the performance of multiple machine learning models; and (3) estimate daytime sleep duration using the resulting sleep–wake classifications. By addressing limitations associated with subjective parent-reported annotations and improving label consistency, this study provides a more reliable and scalable methodology for infant sleep monitoring. The findings demonstrate the feasibility of using wearable accelerometer data and structured label-refinement techniques to improve daytime sleep annotation consistency and machine-learning-based classification performance in infants.

## 2. Related Work

Researchers consistently report systematic discrepancies between parent diaries and objective measures in infants and young children. In 6-month-olds, parent reports overestimated nocturnal sleep relative to actigraphy, with notable disagreement in sleep timing, indicating recall and perception bias in diaries (and motivating calibration to the actigraphic signal) [20]. Similar diary–actigraphy mismatches appear in older pediatric samples and mixed cohorts; for example, a meta-analytic review of pediatric actigraphy documents frequent divergence from diaries in onset, offset, and total sleep time, recommending objective data to validate or correct subjective reports. Preschool/child studies likewise show only moderate agreement among actigraphy, diaries, and questionnaires (often with diaries overestimating sleep), reinforcing the need to reconcile or adjust diary labels using sensor data [21]. In clinical and research practice statements, the AASM emphasizes actigraphy’s value for multi-day, real-world monitoring and cautions that diary entries can be inaccurate—hence the routine practice of aligning diaries to actigraphic rest–activity profiles [22].

Multiple empirical papers explicitly compare, calibrate, or correct diary-based estimates using actigraphy/PSG. In school-aged children, direct comparisons show that objective devices detect more nocturnal awakenings and shorter sleep than diaries, implying overestimation in parent reports and justifying data-driven adjustment [23]. A recent Frontiers study that simultaneously collected child self-report, parent report, and actigraphy found method-dependent discrepancies and advocated integrating actigraphy to refine reported sleep parameters [24]. Infant and toddler reviews similarly conclude that actigraphy (and algorithms derived from it) should be used to validate or correct parental estimates—particularly for naps and sleep timing—because subjective logs miss short, fragmented episodes [25,26,27]. Broader methodological reviews in pediatrics conclude that, while diaries are useful, they require objective corroboration and, where feasible, data-driven correction to reduce misclassification—especially for daytime sleep [24]. Together, these works establish that parent diaries often misestimate infant/child sleep (over-reporting duration; imprecise onset/offset; missed brief naps) and that many studies either formally “adjust” diary labels with actigraphy/PSG or interpret diaries in light of objective data—an approach aligned with our MSW framework’s intent to refine noisy parent-provided labels using sensor-derived thresholds and temporal context.

## 3. Materials and Methods

**Figure 1:**
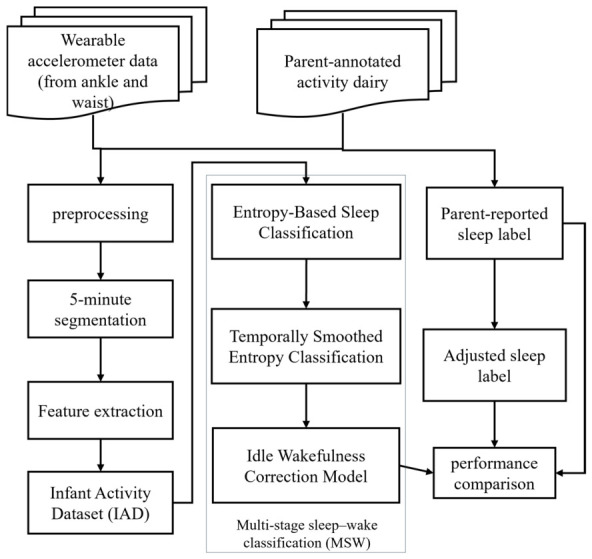
Overview of the methodology.

Figure 1 illustrates the workflow of our sleep–wake classification approach. After collecting raw accelerometer data from ankle and waist devices, the signals were preprocessed and segmented into 5-min epochs. We then applied the MSW method comprising entropy-based thresholding, temporal smoothing, and idle wakefulness correction, followed by performance evaluation using machine learning classifiers.

### 3.1. Data Collection

The data used in this study were obtained with permission from the researchers of a previously published study by Thelagathoti et al. [28]. The data set consists of 13 healthy infants aged between 6 and 15 months. Each infant wore two triaxial accelerometers (GT9X Link, ActiGraph, LLC, Pensacola, FL, USA) [29], which recorded raw acceleration data at a sampling rate of 100 Hz. One device was worn on the right side of the waist, secured with an adjustable elastic belt, while the other was worn on the right ankle, fastened using an elastic band. Data were collected in a free-living environment over a four-day period from 7 am to 7 pm, consisting of two weekdays and two weekend days. Infants were under their typical daily routine throughout the data collection period. Each accelerometer recorded physical activity intensity along the *X*, *Y*, and *Z* axes, as well as the composite vector magnitude (VM). The VM was automatically computed by the device using (1).

(1) $$x=\sqrt{\left(X^{2}+Y^{2}+Z^{2}\right)}$$

Additionally, the dataset includes an activity diary segmented into 30-min intervals. This diary was maintained by parents or caregivers and annotated to indicate periods when the child was reported to be sleeping.

### 3.2. Preparation of Infant Activity Dataset

To facilitate sleep–wake classification, a comprehensive infant activity dataset was prepared by performing multiple steps including data preprocessing, segmentation, feature extraction, and dataset integration. Initially, raw accelerometer data were available as individual CSV files for each infant, with triaxial acceleration readings recorded continuously throughout the day. We merged these raw files for each infant with corresponding parent-reported sleep–wake labels. Parent annotations were logged at a 30-min granularity, indicating whether the infant was asleep or awake. separate files for ankle and waist sensors. These files contained time intervals without parent-reported labels were removed from the dataset to maintain consistency and prevent ambiguity during supervised learning. Given the coarse granularity of the parent diaries, we segmented the data into 5-min intervals to achieve higher temporal resolution. Each 30-min parent-reported label was then uniformly extrapolated to six corresponding 5-min intervals. This finer segmentation allowed us to better capture short, fragmented sleep patterns typical of infant daytime naps.

Next, we extracted relevant features independently from the ankle and waist accelerometer data for each 5-min segment. The features were designed to summarize both movement intensity and temporal variability. These included statistical metrics such as mean, standard deviation (SD), and interquartile range (IQR) of activity. Additionally, we computed active time (number of samples where activity > 0), inactive time (activity = 0), and the signal magnitude area (SMA), a composite feature capturing the overall intensity of motion across all three axes.

Finally, we compiled the processed and segmented data from all infants to generate a single, unified dataset. Each record in this dataset represents a 5-min interval, with corresponding feature values and an extrapolated sleep–wake label. This structured dataset serves as the foundation for training and evaluating sleep classification models.

### 3.3. Multi-Stage Sleep–Wake Classification Using Label Refinement

To address the limitations of noisy and imprecise parent-reported sleep–wake annotations, we developed an MSW framework to systematically refine labels using objective accelerometer-derived information. Parent-reported labels often lack temporal precision and may include inconsistencies due to intermittent observation, motivating the need for data-driven correction. In this framework, empirically derived activity thresholds are used to identify and correct segments where reported labels are inconsistent with observed movement patterns. These thresholds are selected based on the statistical distribution of accelerometer features to capture meaningful distinctions between sleep and wake states. Because threshold-based labeling alone is susceptible to transient fluctuations and motion artifacts, additional temporal smoothing is incorporated to enforce continuity across adjacent segments. Furthermore, a correction step is introduced to account for idle wakefulness, where low-movement wake periods may otherwise be misclassified as sleep. Together, these steps form a structured preprocessing pipeline that generates refined sleep–wake labels, which are subsequently used for model training and evaluation.

#### 3.3.1. Entropy-Based Sleep Classification

To classify infant sleep and wake states, we first applied an entropy-based approach to the extracted accelerometer features using Shannon entropy on the mean of ankle and waist [30]. Entropy quantifies the unpredictability or complexity of a signal and is computed using the following formula in (2).

(2) $$H\left(X\right)=-\sum_{n=1}^{n}\left(p\left(x_{i}\right)\log_{2}p(x_{I})\right)$$

where ${p(x}_{i})\;$ is the probability of signal intensity value $\;x_{i}$ in each 5-min segment. This was computed independently for both ankle and waist sensors over each segment. Low entropy values indicated stable, low-movement states—typical of sleep—while higher entropy suggested active, wakeful states. A sleep threshold *T* was derived empirically from the distribution of entropy values across all subjects for ankle and waist separately. Each segment was classified either as sleep or wake using the formula in (3).

(3) $${State}_{t}=\left\{\begin{array}{c} Sleep,\;\;if\;H_{t}<T\\ Wake,\;\;if\;H_{t}\geq T \end{array}\right.$$

where $H_{t}$ is the entropy at time segment t.

#### 3.3.2. Temporally Smoothed Entropy Classification

To mitigate the impact of transient noise and momentary fluctuations in activity, we applied temporal smoothing to the entropy-based labels. We implemented a majority voting mechanism over a sliding window of three consecutive 5-min segments (i.e., 15 min) [31]. This temporal filter adjusted labels based on the consistency of nearby segments. For each 5-min segment $t_{i}$ we evaluated the window using (4):

(4) $$W_{t}=\{S_{t-1},S_{t},S_{t+1}\}$$

where $S_{t}$ ∈{Sleep, Wake}. The smoothed label $\tilde{S_{t}}$ was determined by (5):

(5) $${\tilde{S}}_{t}=mode(W_{t})$$

For instance, if a single ‘wake’ label was flanked by two ‘sleep’ segments, it was reclassified as ‘sleep’, assuming it was likely a brief movement during a nap. This smoothing process helped to reduce false positives and false negatives, particularly during transitions between sleep and wake states. It ensured more physiologically plausible sequences, aligning the classification closer to real-world infant sleep behavior.

#### 3.3.3. Idle Wakefulness Correction

Despite entropy-based classification and smoothing, a major challenge remained: distinguishing true sleep from “idle wakefulness”—periods when infants are awake but relatively motionless [32]. These episodes often resemble sleep in accelerometer data, leading to misclassification. To address this, we introduced an idle wakefulness correction step that incorporated both signal intensity and temporal context. We analyzed segments originally classified as ‘sleep’ but had sustained low entropy and zero active time across both sensors. If these segments were isolated and not part of a longer continuous sleep bout (e.g., surrounded by wake segments), they were reclassified as ‘wake’. We performed a contextual correction using signal intensity and temporal sequence properties. For each candidate sleep segment $S_{t}$, we computed the Active Time Ratio (*ATR*) as follows mentioned in (6):

(6) $${ATR}_{t}=\frac{{Duration}_{activity>0}}{{Duration}_{total}}$$

Segments where ${ATR}_{t}=0$, and where surrounding segments were classified as wake, were likely idle wakefulness. We reclassified such isolated segments using the following formula in (7):

(7) $${\tilde{S}}_{t}=\left\{\begin{array}{l} Wake,\;\;\;\;\;\;\;\;\;if{\;ATR}_{t}=0\;and\;{\tilde{S}}_{t-1}={\tilde{S}}_{t+1}=Wake\;\\ Sleep,\;\;\;\;\;\;\;\;\;Otherwise \end{array}\right.$$

This correction step helped identify infants who were lying still while awake, thus improving classification accuracy.

Through this three-stage classification pipeline—entropy-based classification, temporal smoothing, and idle wakefulness correction—we generated sleep–wake classification label for the detection of infant sleep with greater precision and reliability. This framework provided a robust foundation for downstream analyses, including sleep duration estimation and comparison with parent-reported data.

## 4. Results

In this section, we present the experimental results evaluating the proposed MSW framework for refining infant sleep–wake annotations from wearable accelerometer data. We first report the outcomes of individual components of the MSW pipeline, including entropy-based classification, temporal smoothing, and idle wakefulness correction. Next, we compare the performance of multiple machine learning models trained using both parent-reported and MSW-derived labels under a leave-one-subject-out cross-validation (LOSO-CV) scheme. Finally, we present total sleep measured for each subject and compare the difference between their sleep on weekdays and weekends.

**Figure 2:**
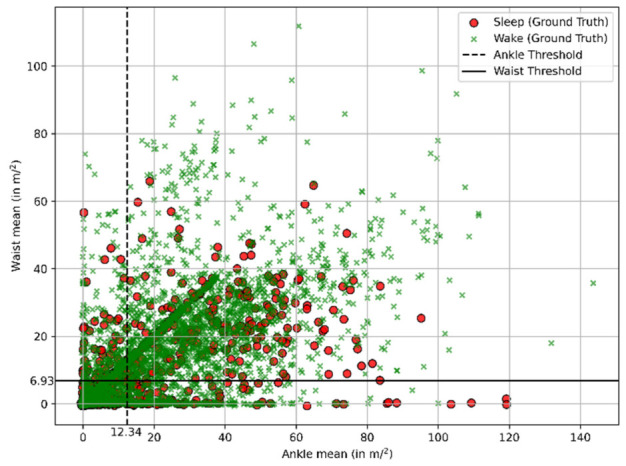
Distribution of parent-reported daytime sleep and wake segments using ankle and waist mean activity derived from raw triaxial accelerometer data (m/s^2^). The ankle (12.34 m/s^2^) and waist (6.93 m/s^2^) activity thresholds were determined using an entropy-based threshold selection method. Red circles denote sleep segments and green crosses denote wake segments. Overlap between classes indicates inconsistencies in parent-reported annotations.

After preprocessing the raw sensor data, first step in MSW approach is to derive empirical separation boundaries for distinguishing sleep and wake states based on movement activity patterns using mean values from ankle and waist accelerometers. Entropy thresholding identifies the point at which the uncertainty (or randomness) in a classification system is minimized, essentially maximizing the information gain. Applying this technique separately to ankle and waist accelerometer data yielded thresholds of 12.34 m/s^2^ for ankle mean and 6.93 m/s^2^ for waist mean. These thresholds are visually indicated in Figure 2 that illustrates the distribution of ground truth daytime sleep and wake segments based on ankle and waist activity levels, highlighting a substantial overlap between the two classes. This overlap reflects the inherent limitation of parent-reported labels, where low-movement wake periods and brief activity during sleep are often misclassified. As seen in Figure 2, several segments labeled as sleep exhibit high activity, while some wake segments fall within low-activity regions, indicating label noise and inconsistency. To address this, entropy-based thresholds (ankle: 12.34 m/s^2^, waist: 6.93 m/s^2^) are introduced to provide an objective separation based on movement intensity, providing an objective basis for refining potentially inconsistent parent-reported annotations.

However, thresholding alone cannot account for transient fluctuations and scattered misclassifications. To reduce the impact of transient noise and sporadic activity, temporal smoothing was applied using a majority voting scheme over a three-segment (15-min) sliding window as explained in Section 3.3.2. For example, a sequence of predicted labels [0,1,0], where a single sleep segment was surrounded by wake segments, was reassigned to [0,0,0], reflecting a more physiologically plausible wake state. Conversely, a sequence [1,0,1] was corrected to [1,1,1], reducing false wake detections during sustained sleep. This smoothing step significantly reduced rapid oscillations in the sleep–wake labels and improved temporal consistency. Despite temporal smoothing, some isolated sleep segments remained, primarily due to idle wakefulness, where infants were awake but exhibited minimal movement. To address this, a correction rule was applied to reclassify isolated sleep points. For instance, if a smoothed sequence contained [0,1,0], where the middle segment was labeled as sleep but both neighboring segments were wake, the central segment was reclassified as wake. This correction eliminated false-positive sleep detections caused by motionless wake behavior, further refining the final sleep–wake labels and improving overall classification reliability.

After applying MSW approach, we evaluated the impact of post-processing steps on original parent-reported sleep labels (as shown in Table 1). Initially, entropy-based thresholding resulted in a 38.34% deviation from the parent-reported labels. Temporal smoothing corrected 3.69% of these predictions, followed by a minor adjustment of 0.15% through idle wakefulness correction. Overall, 37.52% of the final sleep labels differed from the original parent labels (Table 1). In this table, each epoch represents a 5-min segment. These changes reflect the degree to which the MSW framework refined the original parent annotations and should not be interpreted as definitive corrections in the absence of an independent reference standard.

To evaluate the impact of the MSW label-refinement process, multiple machine learning models were trained and evaluated using two annotation schemes: the original parent-reported labels and the MSW-derived proxy sleep–wake labels (Table 2). Sleep epochs were treated as the positive class and wake epochs as the negative class for all classification analyses. Model performance was assessed using a leave-one-subject-out cross-validation (LOSO-CV) scheme to ensure subject-independent evaluation and robust generalization across infants. For each LOSO-CV fold, performance metrics (accuracy, sensitivity, specificity, F1 score, and AUC) were calculated on the held-out subject. The reported values correspond to the mean performance across all CV folds. The corresponding 95% confidence intervals were calculated as mean ± 1.96 × (SD/√*n*), where *n* denotes the number of CV folds. AUC values were computed using classifier prediction probabilities generated for the held-out subject in each CV fold and subsequently averaged across folds. When trained using parent-reported labels, all models exhibited lower sensitivity, reflecting a strong bias toward predicting wakefulness, likely due to label noise, class imbalance, and inconsistencies in parent-reported sleep annotations. For example, Logistic Regression achieved a sensitivity of 0.073, and SVM achieved 0.101, despite relatively high specificity. In contrast, models trained and evaluated using MSW-derived labels achieved better performance across all evaluation metrics. Notably, the Random Forest classifier achieved an accuracy of 0.969, sensitivity of 0.966, specificity of 0.969, F1 score of 0.963, and AUC of 0.988 using MSW-derived labels, compared to an accuracy of 0.676 and sensitivity of 0.189 when trained with parent-reported labels. Similar improvements were observed across other models. Logistic Regression improved from an F1 score of 0.040 (parent) to 0.948 (MSW), while SVM showed a substantial increase in AUC from 0.584 to 0.979.

It is important to note that the parent-reported labels and MSW-derived labels represent two distinct annotation schemes rather than a common external ground truth. Parent-reported labels were obtained independently from caregivers and therefore provide an external annotation source, although they may contain temporal imprecision and reporting inconsistencies. In contrast, the MSW-derived labels were generated using the same accelerometer recordings subsequently used for feature extraction and model development. Consequently, the performance metrics reported for the MSW-derived labels should be interpreted as model agreement with the refined proxy annotation framework and not as independent validation of true infant sleep–wake states. The observed differences between parent-reported and MSW-derived results therefore reflect differences between the respective annotation schemes rather than differences in validated sleep-detection accuracy.

**Figure 3:**
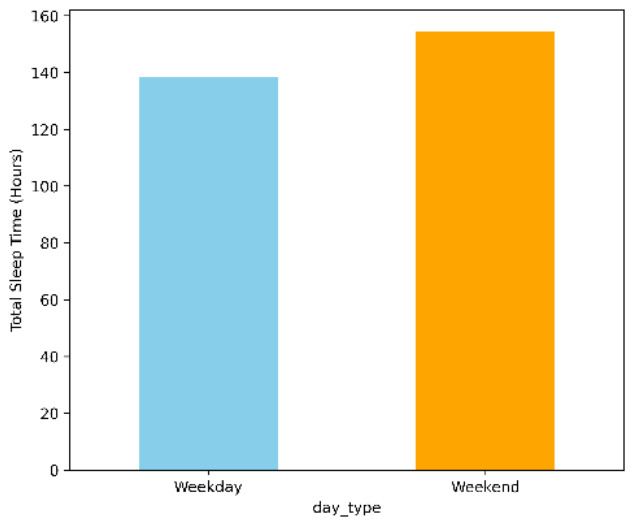
Total daytime sleep duration between weekday and weekend.

**Figure 4:**
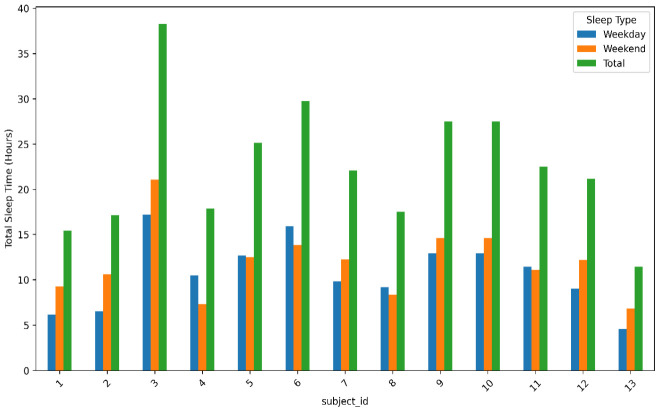
Daytime sleep duration of each infant on weekday, weekend, and total sleep duration.

Using the final sleep–wake labels derived from MSW framework, we estimated the total estimated daytime sleep duration (in hours) for each subject across weekdays and weekends. The results shown in Figure 3 and Figure 4 highlight both inter-individual variability and day-type differences in sleep patterns. Across 13 subjects, the total cumulative weekday sleep duration was 138 h, while weekend sleep duration totaled 154 h, indicating a cumulative difference of approximately 16 h within this cohort. Because no formal statistical testing was performed and the sample size was limited, these observations should be interpreted descriptively rather than as evidence of significant weekday–weekend sleep differences. On an individual level, subjects 1 and 2 exhibited the largest differences between weekday and weekend sleep, with subject 2 showing a 4.08-h increase on weekends (6.5 h vs. 10.58 h). Conversely, subject 4 showed a slight decrease in weekend sleep (10.5 h on weekdays vs. 7.33 h on weekends), possibly indicating irregular patterns or external influences. Subjects 9 and 10 had identical sleep durations, showing a consistent increase in weekend sleep by 1.67 h each.

The observed differences between the parent-reported and MSW-derived annotation schemes suggest that the MSW framework can be used as a systematic label-refinement methodology for reducing inconsistencies in caregiver-reported sleep annotations. By integrating activity-based thresholding, temporal smoothing, and idle wakefulness correction, the framework generates refined proxy sleep–wake labels that may support future wearable-based infant sleep studies when reliable sleep annotations are unavailable or difficult to obtain.

## 5. Discussion

Accurate assessment of infant sleep remains a significant challenge, particularly for daytime sleep, which is short, fragmented, and difficult for caregivers to monitor continuously. Parent-reported sleep diaries are commonly used in both research and clinical settings; however, their coarse temporal resolution and susceptibility to recall bias limit their reliability as sleep annotations for training and validating sensor-based sleep detection algorithms. The findings of this study demonstrate that the proposed MSW framework effectively addresses these limitations by leveraging objective accelerometer-derived information to refine noisy labels and improve downstream model performance.

A key contribution of the MSW approach is its ability to generate refined proxy sleep–wake annotations in the absence of reliable annotations. When models were trained using parent-reported labels, performance was consistently constrained by low sensitivity, indicating a strong bias toward predicting wakefulness and an inability to reliably identify sleep episodes. This issue was particularly evident under subject-independent evaluation, where inter-infant variability further amplified label noise. In contrast, MSW-derived labels produced balanced improvements across accuracy, sensitivity, specificity, F1 score, and AUC for all evaluated models. These results suggest that the MSW framework reduces inconsistencies in parent-reported annotations and generates labels that are more consistent with underlying movement patterns derived from accelerometer signals.

The multi-stage design of the MSW pipeline plays a critical role in its robustness. Entropy-based classification enables the detection of low-activity sleep states from noisy accelerometer signals, while temporal smoothing enforces continuity and reduces spurious transitions caused by brief movements. The idle wakefulness correction further addresses a well-known limitation of actigraphy by distinguishing true sleep from motionless wake periods. Together, these stages systematically reduce label noise without relying on manual intervention or external clinical measurements, making the approach scalable and practical for real-world deployments.

From an application perspective, the MSW framework is particularly valuable in settings where parent-reported data are missing, inconsistent, or unreliable—such as in longitudinal home monitoring, daycare environments, or studies involving multiple caregivers. By enabling automated refinement of sleep–wake annotations using wearable sensor data, this method supports continuous, unobtrusive monitoring of infant sleep behavior. However, the MSW-derived labels should be interpreted as refined proxy annotations rather than clinically validated ground truth, as independent validation against PSG or expert behavioral coding was not available in the current study.

Furthermore, our findings align with previous studies that have identified discrepancies in parent-reported sleep data. Research has shown that parents often overestimate their children’s sleep duration and may misclassify wake periods as sleep [33,34,35,36]. For example, parent-reported sleep logs often suffer from recall bias and misclassification, especially in infants and toddlers whose sleep episodes are fragmented or irregular [37,38]. Werner et al. reported discrepancies between actigraphy and parental diaries, particularly in detecting brief night awakenings [14]. Similarly, Spruyt and Gozal found that overestimation of total sleep time is common in subjective reports [10]. The substantial divergence observed between parent-reported and MSW-derived annotations in our dataset highlights the need for systematic label-refinement approaches when using caregiver-reported sleep information in wearable-based sleep studies.

## 6. Limitations and Future Directions

While our study introduces a novel MSW classification framework and demonstrates its potential to enhance sleep–wake annotation refinement in infants using wearable accelerometry, several limitations should be considered. The MSW framework, though effective in correcting noisy parent-reported labels, has not been validated against gold-standard tools such as polysomnography (PSG), leaving uncertainty about the clinical accuracy of the derived labels. Therefore, the MSW-derived labels should be interpreted as refined proxy annotations rather than clinically validated ground truth. The limited sample size also restricts the generalizability of findings across diverse pediatric populations. Furthermore, the reliance on parent-reported sleep annotations as the initial reference introduces potential biases due to subjective interpretation and recall errors. Because the MSW-derived labels were generated from the same accelerometer recordings used for downstream model development and evaluation, some degree of circularity may exist between the label-refinement process and classifier performance. Consequently, the reported performance metrics should be interpreted as agreement with the refined annotation framework rather than independent validation of true sleep–wake states. Future work should validate MSW-derived labels against PSG or actigraphy paired with detailed sleep diaries, expand the participant pool across varied age groups and demographics, and explore the integration of contextual factors like light exposure, feeding, and environmental conditions. Furthermore, entropy thresholds were empirically derived from the global distribution of entropy values across the dataset and used as fixed population-level parameters throughout the MSW pipeline. These thresholds were not optimized using model performance or test labels. We acknowledge that this approach may introduce limited information leakage under LOSO-CV, and future work will investigate fold-specific threshold estimation using training subjects only.

## 7. Conclusions

This study presents a robust multi-stage workflow (MSW) for improving the consistency of infant sleep–wake annotations using wearable accelerometer data. By correcting noisy parent-reported labels through entropy-based thresholding, temporal smoothing, and idle wakefulness correction, the MSW framework generated more internally consistent proxy sleep–wake annotations. Comparative analyses showed that machine learning models trained and evaluated using MSW-derived labels achieved better performance metrics than those trained and evaluated using the original parent-reported labels, highlighting the influence of annotation quality and consistency on downstream model performance. The study underscores the value of automated, objective tools like MSW in pediatric sleep research and demonstrates the feasibility of using wearable sensor data to refine noisy sleep annotations in naturalistic settings. Importantly, the parent-reported labels remain the only independent caregiver-provided annotations available in this study, whereas the MSW-derived labels were generated from the same accelerometer recordings used for model development. Therefore, the reported performance metrics should be interpreted as results relative to the respective annotation schemes rather than independent validation of true infant sleep–wake states. While the MSW-derived labels have not been validated against an independent gold standard, the findings contribute to a growing body of research exploring label-refinement approaches for improving the consistency of wearable-based sleep analyses and supporting future infant sleep monitoring studies. Furthermore, the proposed MSW framework may serve as a scalable label-refinement methodology for generating more consistent proxy sleep–wake annotations in wearable-sensor datasets where reliable sleep labels are unavailable or difficult to obtain.

## Figures and Tables

**Table 1:** Change in parent-reported sleep labels after applying MSW approach.

Label Transition Stage	Changed Epochs	Percentage
Parent Label → Entropy-Based Sleep Classification	2319	38.34%
Entropy-Based Sleep Classification → Temporally Smoothed Entropy Classification	223	3.69%
Temporally Smoothed Entropy Classification → Idle Wakefulness Correction	9	0.15%
Parent Label → Idle Wakefulness Correction (Overall Change)	2269	37.52%

**Table 2:** Model performance results using parent-reported labels and MSW-derived proxy labels under leave-one-subject-out cross-validation (LOSO-CV). Values are reported as mean ± 95% confidence interval (standard deviation). Sleep epochs were treated as the positive class. The reported metrics should be interpreted relative to the respective annotation schemes and do not represent independent validation of true sleep–wake states.

Model	Label Type	Accuracy	Sensitivity	Specificity	F1 Score	AUC
Logistic Regression	Parent	0.654 ± 0.074 (0.123)	0.073 ± 0.150 (0.250)	0.954 ± 0.092 (0.153)	0.040 ± 0.071 (0.118)	0.687 ± 0.079 (0.132)
	MSW	0.955 ± 0.011 (0.018)	0.948 ± 0.019 (0.031)	0.955 ± 0.013 (0.022)	0.948 ± 0.016 (0.027)	0.989 ± 0.004 (0.007)
Random Forest	Parent	0.676 ± 0.071 (0.119)	0.189 ± 0.056 (0.094)	0.921 ± 0.018 (0.030)	0.265 ± 0.072 (0.120)	0.669 ± 0.064 (0.106)
	MSW	0.969 ± 0.012 (0.020)	0.966 ± 0.017 (0.028)	0.969 ± 0.012 (0.021)	0.963 ± 0.018 (0.030)	0.988 ± 0.007 (0.011)
Decision Tree	Parent	0.610 ± 0.055 (0.091)	0.234 ± 0.055 (0.091)	0.797 ± 0.039 (0.065)	0.269 ± 0.054 (0.090)	0.595 ± 0.038 (0.064)
	MSW	0.946 ± 0.018 (0.031)	0.934 ± 0.029 (0.048)	0.948 ± 0.018 (0.030)	0.934 ± 0.027 (0.045)	0.928 ± 0.022 (0.037)
SVM	Parent	0.631 ± 0.076 (0.126)	0.101 ± 0.162 (0.270)	0.917 ± 0.119 (0.199)	0.051 ± 0.076 (0.128)	0.584 ± 0.061 (0.101)
	MSW	0.959 ± 0.010 (0.017)	0.957 ± 0.015 (0.025)	0.955 ± 0.013 (0.021)	0.953 ± 0.015 (0.026)	0.979 ± 0.008 (0.014)
XGBoost	Parent	0.667 ± 0.067 (0.112)	0.208 ± 0.055 (0.093)	0.895 ± 0.021 (0.036)	0.277 ± 0.063 (0.105)	0.664 ± 0.064 (0.107)
	MSW	0.965 ± 0.014 (0.023)	0.959 ± 0.020 (0.033)	0.965 ± 0.012 (0.021)	0.957 ± 0.020 (0.034)	0.988 ± 0.006 (0.010)

## Data Availability

The data presented in this study are available on request from the corresponding author. The data are not publicly available due to privacy.
